# Exploring novel algorithms for atrial fibrillation detection by driving graduate level education in medical machine learning

**DOI:** 10.1088/1361-6579/ac7840

**Published:** 2022-07-07

**Authors:** Maurice Rohr, Christoph Reich, Andreas Höhl, Timm Lilienthal, Tizian Dege, Filip Plesinger, Veronika Bulkova, Gari Clifford, Matthew Reyna, Christoph Hoog Antink

**Affiliations:** 1 KIS*MED — AI Systems in Medicine, Technische Universität Darmstadt, Darmstadt, Germany; 2 Institute of Scientific Instruments of the Czech Academy of Sciences, Brno, Czech Republic; 3 Medical Data Transfer, s.r.o., Brno, Czech Republic; 4 Department of Biomedical Informatics, Emory University, United States of America; 5 Department of Biomedical Engineering, Georgia Institute of Technology, United States of America

**Keywords:** gamification, atrial fibrillation, electrocardiogram, deep learning

## Abstract

During the lockdown of universities and the COVID-Pandemic most students were restricted to their homes. Novel and instigating teaching methods were required to improve the learning experience and so recent implementations of the annual PhysioNet/Computing in Cardiology (CinC) Challenges posed as a reference. For over 20 years, the challenges have proven repeatedly to be of immense educational value, besides leading to technological advances for specific problems. In this paper, we report results from the class ‘Artificial Intelligence in Medicine Challenge’, which was implemented as an online project seminar at Technical University Darmstadt, Germany, and which was heavily inspired by the PhysioNet/CinC Challenge 2017 ‘AF Classification from a Short Single Lead ECG Recording’. Atrial fibrillation is a common cardiac disease and often remains undetected. Therefore, we selected the two most promising models of the course and give an insight into the Transformer-based DualNet architecture as well as into the CNN-LSTM-based model and finally a detailed analysis for both. In particular, we show the model performance results of our internal scoring process for all submitted models and the near state-of-the-art model performance for the two named models on the official 2017 challenge test set. Several teams were able to achieve F_1_ scores above/close to 90% on a hidden test-set of Holter recordings. We highlight themes commonly observed among participants, and report the results from the self-assessed student evaluation. Finally, the self-assessment of the students reported a notable increase in machine learning knowledge.

## 1. Introduction

As a result of the COVID-Pandemic and the lockdown of universities, novel teaching concepts combining online teaching, experimenting, and self-learning with a stimulating environment are needed. Challenge-based **gamification** aspects such as clear tasks, leaderboards, instant feedback, and a scoring system showed promising results in improving statistics and engineering education in recent studies (Legaki *et al* 2020, Colombari *et al* 2021). In particular, leaderboards offer a system of self-feedback and goal-setting to students (Nah *et al* 2014). It comes to no surprise that the annual PhysioNet/Computing in Cardiology (CinC) Challenges not only lead to technological advances for specific problems, they also repeatedly proved to be of immense educational value for participants. Inspired by the PhysioNet/CinC Challenge of 2017 ‘AF Classification from a Short Single Lead ECG Recording’ (Clifford *et al* 2017), we developed the project seminar ‘Artificial Intelligence in Medicine Challenge’ as part of the electrical/biomedical engineering curriculum at TU Darmstadt with the goal to detect **atrial fibrillation** in one-lead electrocardiograms (ECGs). Atrial fibrillation (AFib) is the most common sustained cardiac arrhythmia (Lip *et al* 2016) and therefore of major interest for regular screening. We set up our own system for running models for AFib detection developed by the participants, inferring predictions and scoring these predictions respectively. Since research (Toda *et al* 2017) has shown that gamification alone may not be the ‘holy grail of education’, we tried to counteract potential negative effects. These negative effects include demotivation due to excessive competition and a focus shift from creative development to the gamification mechanics. In particular, we offered weekly sessions to discuss ideas, checked problems with the teams, and had a voluntary mid-semester presentation about intermediate results. Consequentially, we made clear from the start that the originality of approaches and a good analysis were crucial to us and that a good score and ranking was neither required nor sufficient for a good grade. Hence, the award for the winning team consisted of a certificate and a small price but was not tied to the grade. The aim of this paper is thus to present an innovative course design for machine learning education and to show that novel deep learning models developed in the course of the project are competitive in AFib detection. In order to show detailed analysis of the performance and novelty of these models, we evaluated them on the original PhysioNet 2017 challenge test set and compared them to the winning models of the challenge of 2017 (Datta *et al* 2017, Zabihi *et al* 2017, Teijeiro *et al* 2018).

Compared to our conference paper (Rohr *et al* 2021), we made significant changes and added the following unreported results. We show that models designed by the participants achieve near state-of-the-art performance in atrial fibrillation classification. This includes results for the multilabel classification on the unseen dataset of the PhysioNet Challenge and deeper insights into the models. Moreover, we show that using gamification concepts in machine learning education additionally motivates participants which results in competitive models. At the point of writing this paper the course is already in its second iteration and we cover the implementation of some of the changes inspired by the evaluation and share code and documentation of our evaluation system.

## 2. Methods

We provided recordings of the ECG from multiple data sources and measurement devices to the participants of the course, split into subsets. While the ECG recordings were classified into the four categories **normal sinus rhythm** (‘NSR’), **atrial fibrillation** (‘AFib’), **other rhythm** (‘Other’) and **noisy recording** (‘Noisy’) in the original challenge, the main task for our class was only to distinguish ‘AFib’ from ‘NSR’. The secondary goal was to correctly classify all four classes (table 1). We deployed a system consisting of a validation phase where each team was allowed 5 successful entries and a final test of the models such that no information about the test data might leak into the model design process. We analyzed the model architecture and signal processing choices of all teams quantitatively. Finally we selected two high performing models with novel architectures (section 2.3) and optimized them for the four class task and evaluated them on the original PyhsioNet/CinC Challenge 2017 test set.

**Table 1.** Relevant labeled recordings for each tasks.

Label	Main task	Secondary task
NSR	✓	✓
AFib	✓	✓
Other		✓
Noisy		✓

### 2.1. Datasets

**Figure 1.**
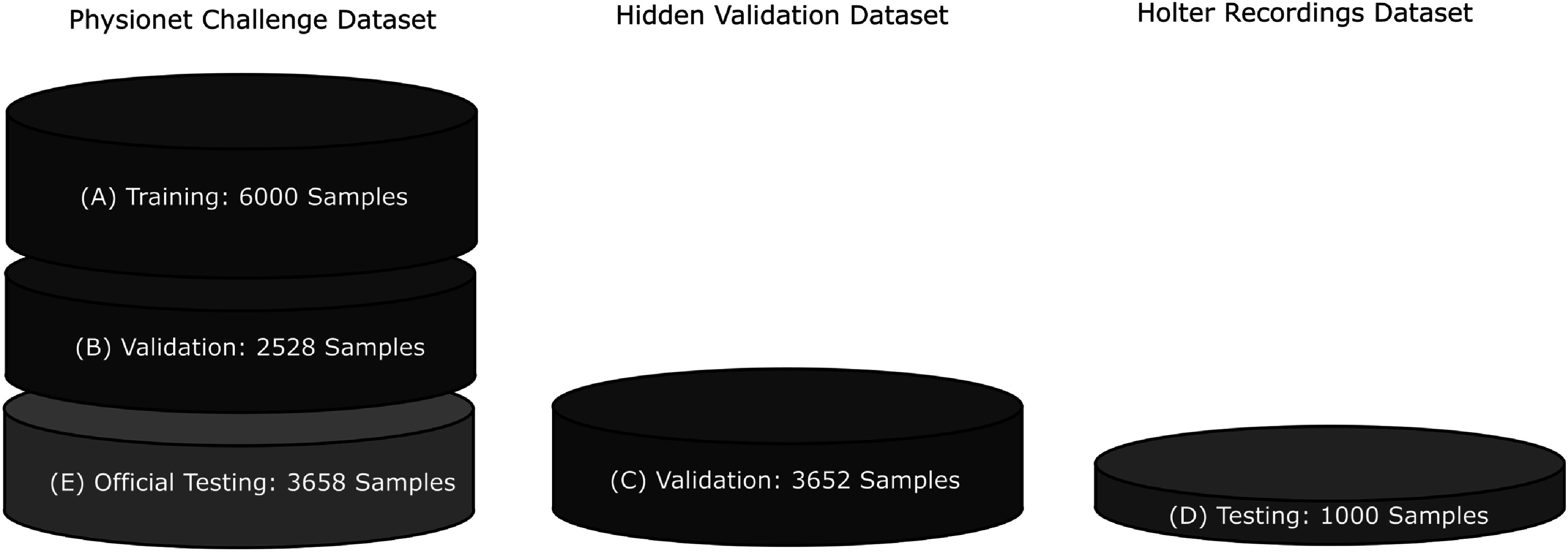
We split the data we acquired into three types of datasets. The color for each dataset codes the phase in which the data is used.

In order to achieve meaningful evaluation results and teach clean data management, we split the data we acquired into *training*, *validation*, and *test* sets (figure 1). Additionally, to render things more realistic and hence more ‘interesting’, the datasets were not drawn from the same distribution but were recorded with different measurement systems. In the following description of the datasets, we also included the official PyhsioNet/CinC Challenge 2017 test set (Clifford *et al* 2017), which was used externally to verify and compare the performance of the two best participant models to the state of the art.

**A** (training) From the official CinC 2017 training set (Clifford *et al* 2017), 6000 randomly selected samples were handed out as training set. The recordings are short single-channel ECGs from the AliveCor device. Four classes [‘NSR’, ‘AFib’, ‘Other’, ‘Noisy’] are available.
**B** (validation) 2528 remaining samples held back from the official CinC 2017 training set. Four classes [‘NSR’, ‘AFib’, ‘Other’, ‘Noisy’] are available.
**C** (validation) A ‘quasi hidden’ validation set was sampled from an openly-available ECG-database containing 3652 examples. Three classes [‘NSR’, ‘AFib’, ‘Other’] are available.
**D** (testing) A ‘true hidden’ set was provided by Medical Data Transfer, s.r.o. Brno, Czechia, containing 1000 Holter recordings during patients daily activities with two classes [‘NSR’, ‘AFib’].
**E** (testing external) 3658 samples of the official CinC 2017 test set. Four classes [‘NSR’, ‘AFib’, ‘Other’, ‘Noisy’] are available.

Besides these, we also used the Icentia11k dataset (Tan *et al* 2019) and the MIT-BIH Arrhythmia Database (Moody and Mark 2001) during training and validation of the proposed novel models.

### 2.2. Set-up of the course

**Figure 2.**
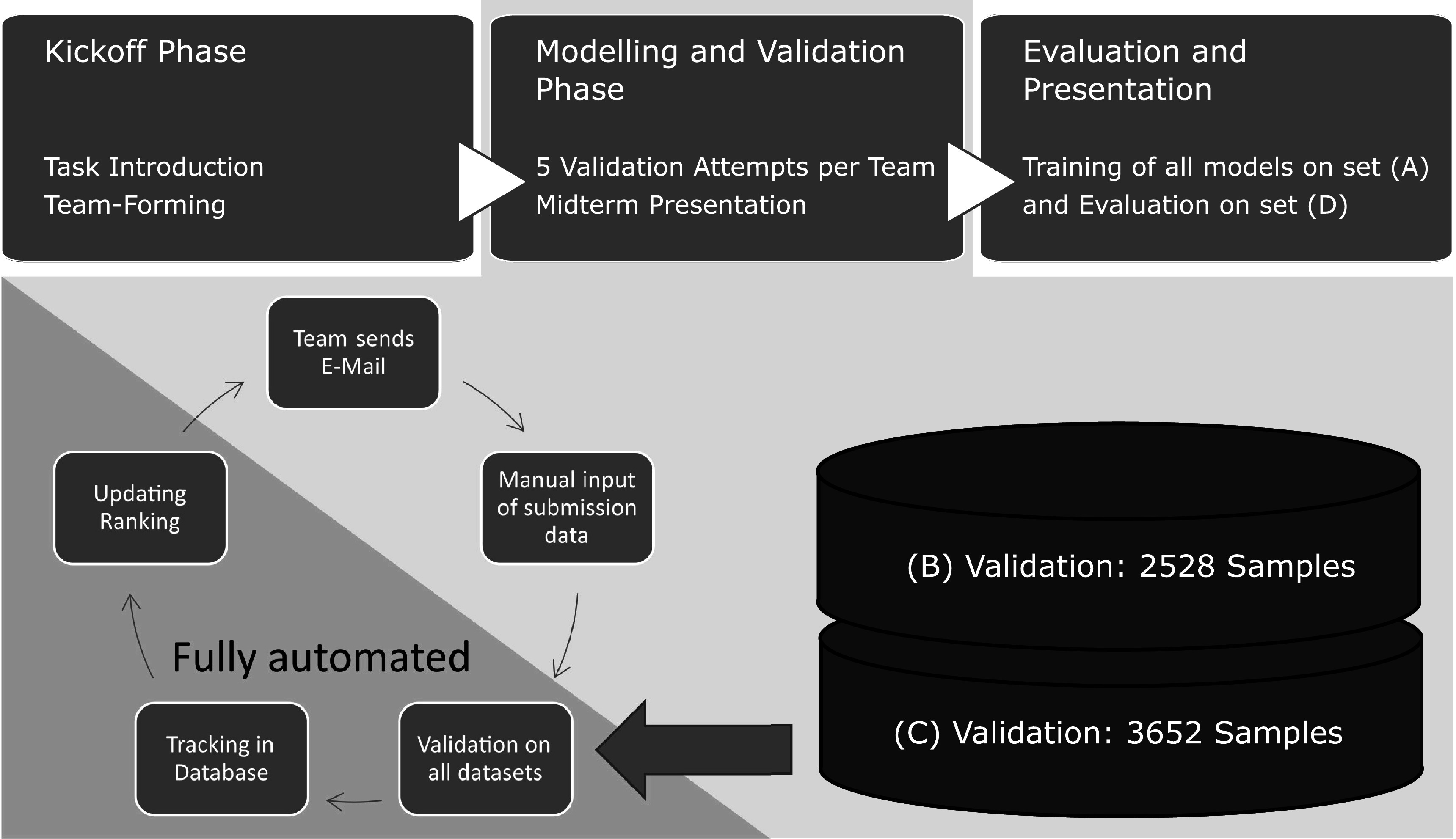
The flow of the project seminar is on the top, below the validation phase is detailed. In the beginning students form teams of up to 3 people and work iteratively on their models with validation steps. The test set is only used in the final evaluation.

The participants were instructed about the problem in a kick-off video meeting. A simple **example code** (‘KIS*MED Model’) was provided to the participants in the form of a jupyter-notebook^6^ 
^6^
Google Colab: https://colab.research.google.com/drive/1AoloKP-ZfZ7rRJu6-aq1cG1PkJHS7KJS (in German).. The model was meant as a baseline and an easy starting point to explore more sophisticated methods. It exploits that AFib is often characterized by irregular beat-to-beat intervals (BBIs) (Couceiro *et al* 2008) and simply computes the BBIs from detected QRS-complexes and classifies the training data based on a threshold on the standard deviation of BBIs. The KIS*MED Model was also provided as a reference implementation^7^ 
^7^
KIS*MED on GitHub: https://github.com/KISMED-TUDa/18-ha-2010-pj. for the interface to our scoring system and provided example python-files for training and inference from the model, as well as standard functionality to compute the score, load in the data, and save predictions. Figure 2 shows the more general flow of the course, with kickoff, a 3 month modelling and validation phase, and the final evaluation of the models. The participants were asked to form groups of 2–3 members or alternatively were grouped by us. We offered a weekly video-meeting during the validation phase, where teams could discuss their main problems and ask questions. After roughly 2 months, all teams presented their general ideas and the difficulties they were facing. ‘Tricks’ used for achieving good scores were mostly kept secret at that time.

During the validation phase, each team was allowed 5* model submissions*, only counting successful runs. Model code was provided via git repositories and processed semi-automatically in order to check if the information given was consistent. Evaluation was performed in python environments^8^ 
^8^
All performed in-house on a Ubuntu Linux based system with two NVIDIA Quadro RTX5000 GPUs and two Intel Xeon @ 3.8 GHz and 256 GB RAM. by running the inference code for each team. A **SQL database** was used to store the evaluation metrics, team information, dataset information, and all information about the validation runs such as run time, console outputs, confusion matrices. After each evaluation, the instantaneous ranking was updated based on this data. Only the models of the final submissions were also trained on our system to check if the performance is plausible.

The participants were encouraged to use their own PCs or other free GPU computing resources such as *Google Colab* and were given access to the TU Darmstadt Lichtenberg high performance computer (HPC), which provides high performance parallel computing capabilities. A short introduction to the usage of the HPC and parallel computing on a batch system was given, but to the best of our knowledge, only one team made significant use of the HPC.

### 2.3. Deep learning models

**Figure 3.**
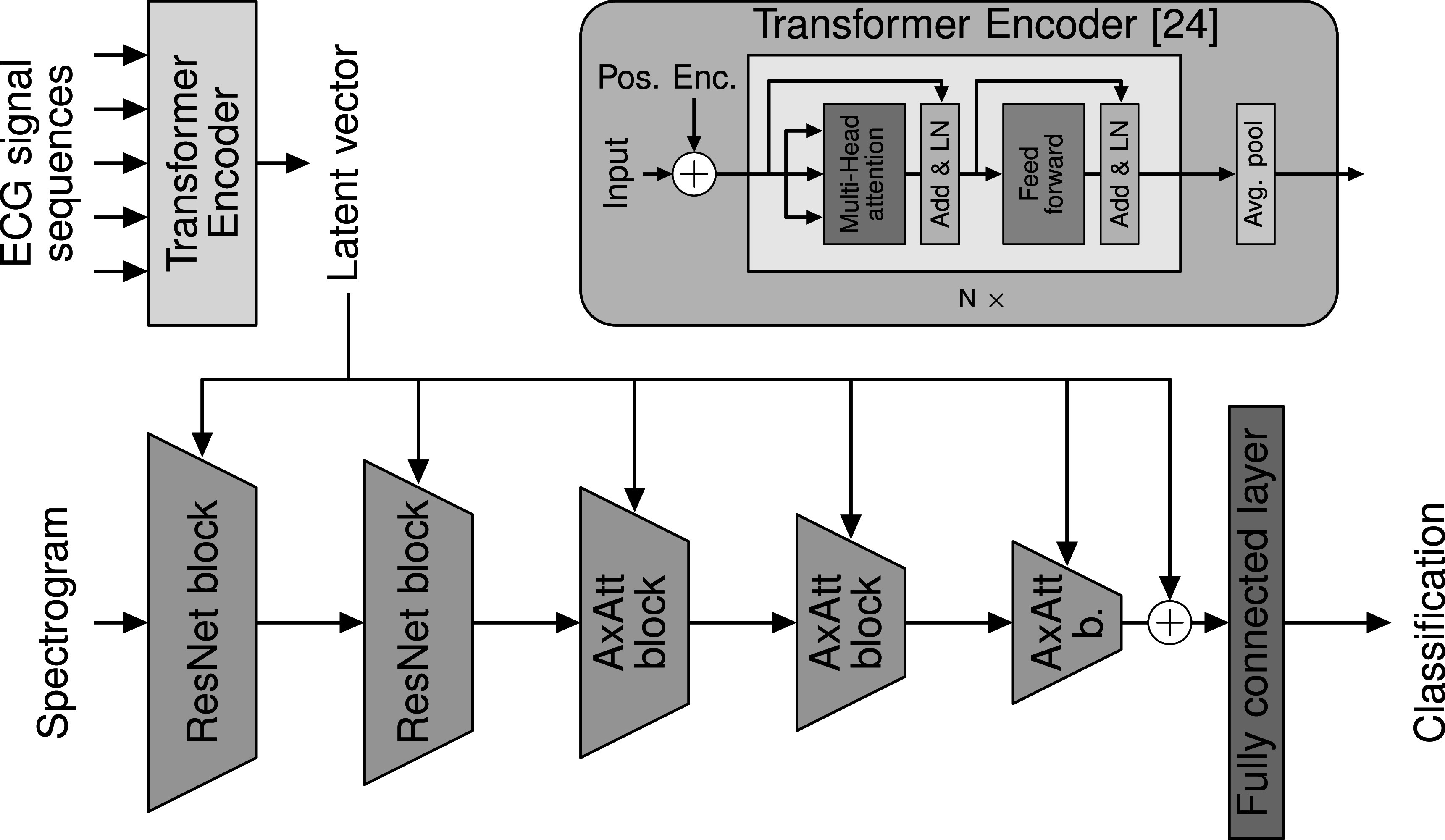
ECG-DualNet++ architecture with spectrogram and ECG signal as inputs. The ECG signal sequence gets encoded by a Transformer encoder (in yellow 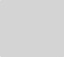 ) to a single latent vector. The spectrogram is encoded by the spectrogram encoder (in green 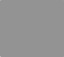 ) composed of two ResNet (He *et al* 2016) blocks and three Axial-Attention (Wang *et al* 2020) blocks. All spectrogram encoder blocks incorporate the latent vector with Conditional Batch Normalization (de Vries *et al* 2017). The classification prediction is obtained by a final fully connected layer with softmax activation (in violet 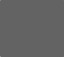 ). Transformer encoder (Vaswani *et al* 2017) architecture shown in the top right.

**Figure 4.**
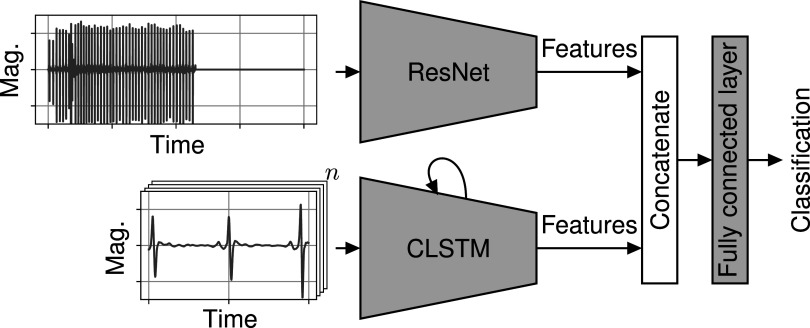
The ECG-RCLSTM architecture with a ResNet on the left and a CLSTM on the right. The signal is zero-padded and fed as a whole into the ResNet. For the CLSTM we split the signal into two second long beats around the R-Peaks. The results of both architectures are then concatenated into a fully connected linear layer to produce a classification.

Recent work on detecting cardiac abnormalities in ECG signals with **deep neural networks** focused on using either input data in the time or the frequency-domain (Zihlmann *et al* 2017, Mashrur *et al* 2019, Khriji *et al* 2020). We present two different deep neural network models, **ECG-RCLSTM-Net** and **ECG-DualNet**, which both make use of a convolutional neural network (CNN), residual network (ResNet)(He *et al* 2016) and long short term memory (LSTM)(Hochreiter and Schmidhuber 1997). ECG-RCLSTM-Net consists of two main building blocks: a ResNet which takes the whole signal as an input and is designed to analyse global features and a CNN-LSTM architecture (CLSTM) (Shi *et al* 2015, He *et al* 2016, Hong *et al* 2020, Xiaolin *et al* 2020) which analyses local features by focusing on segmented beats as shown in figure 4. Inspired by the ECGNET (Mousavi *et al* 2019) and recent advances in deep learning, we present **ECG-DualNet**, a novel neural network architecture for single-lead ECG classification that utilises input data in both the time and frequency-domain. This enables ECG-DualNet to learn features from the time and frequency-domain, redressing the need for engineered input features (Linschmann *et al* 2021). Based on the same global architecture as ECG-DualNet we also present ECG-DualNet++ (figure 3). For ECG-DualNet++, we substitute CNN and LSTM blocks with recent state-of-the-art deep learning building blocks, such as Transformers (Vaswani *et al* 2017) and Axial-Attentions (Wang *et al* 2020).

#### 2.3.1. Network architectures

In **ECG-RCLSTM-Net** same-length ECG recordings are put into the ResNet block. Beat-segments are first fed into a 1D-CNN to obtain a feature representation and then fed into an LSTM. Finally, the outputs of the two networks are concatenated and fed into a fully connected classification layer.

The global network architecture of the **ECG-DualNet(++)**, as presented in figure 3, comprises a signal encoder and a spectrogram encoder. The time-domain ECG signal, cropped into short sequences, builds the input to the signal encoder. Based on this input a latent vector is predicted. The spectrogram of the ECG signal is fed into the spectrogram encoder. Similar to image encoders (He *et al* 2016, Huang *et al* 2017, Reich *et al* 2021), which reduce the spatial resolution of image features, the spectrogram encoder reduces the dimension of the frequency-domain features in each block. Conditional batch normalization (de Vries *et al* 2017) (CBN), used in each spectrogram encoder block, integrates the latent vector of the signal encoder to the frequency-domain features. The ECG-DualNet employs a standard LSTM as the signal encoder and five ResNet blocks with CBN for the spectrogram encoder. The ECG-DualNet++ utilises a Transformer encoder as the signal encoder. Two ResNet blocks followed by three Axial-Attention blocks, all with CBN, build the spectrogram encoder.

#### 2.3.2. Preprocessing

In order to counteract imbalances in the training data, we oversample the datasets by copying individual recordings from the minority class and adding them to the training dataset until the classes are balanced. Fundamentally, the preprocessing is composed of four steps: data augmentation, filtering/transformation, standardization, padding.

The data augmentation pipeline applies a variety of data augmentations to the ECG signal randomly. This improves the generalization of the trained network and prevents overfitting (Perez and Wang 2017, Hatamian *et al* 2020, Nonaka and Seita 2020). The following augmentations are used: *dropping, cut-out, resampling, random resampling, scaling, shifting, sine addition, artificial noise, window warping and bandpass filtering*. The dropping augmentation sets random samples of the ECG signal to zero, the cut-out augmentation sets a random sequence to zero. In the resampling augmentation, the whole signal is resampled to emulate a different heart rate. Random resampling is inspired by the random elastic deformation (Simard *et al* 2003, Ronneberger *et al* 2015, Prangemeier *et al* 2020) used for image augmentation. The ECG signal is resampled by applying smooth random offsets, resulting in a changing heart rate. In the scaling augmentation, the signal gets scaled by a random factor. The sine addition augmentation adds a sinusoidal signal with a random magnitude and phase to the ECG signal. The shift augmentation shifts the ECG signal by a random length. The artificial noise augmentation adds Gaussian noise to the original recordings. Window warping means that we randomly adjust the sampling frequency of randomly selected windows. Finally, in the band pass filter augmentation, the ECG signal is filtered by a band-pass.

For ECG-RCLSTM-Net we confine augmentation to *artificial noise, resampling and window warping*. After augmentation, the data is filtered using a finite impulse response (FIR) bandpass filter, to remove noise from the training data. Finally, each lead is normalized using its mean and standard deviation. To obtain isolated heart beats, we are using the hamilton segmenter (Hamilton 2002). In order to retain the information about the distances of the individual R-Peaks, we include one second before and after each R-Peak. Finally, the input signal of the ResNet is zero padded.

Conversely for ECG-DualNet, the augmented ECG signal is directly standardized to zero mean and unit variance. In the third step, the log spectrogram of the ECG signal is computed with a window length of 64, a hop size of 32, and 64 bins. Recent work showed, using the logarithmic spectrogram improves the classification accuracy of CNN’s (Zihlmann *et al* 2017). Finally, both the ECG signal and the spectrogram are zero-padded to a fixed length.

#### 2.3.3. Training approach and datasets

We train both models in an end-to-end manner on a weighted version of the cross-entropy loss (Goodfellow *et al* 2016)

\begin{eqnarray*}{ \mathcal L }=-\displaystyle \frac{1}{N}\sum _{j=1}^{N}\sum _{i=1}^{C}{\alpha }_{i}\,{y}_{{ji}}\,\mathrm{log}({\hat{y}}_{{ji}}),\end{eqnarray*}

where *C* is the number of classes, ${{\bf{y}}}_{j}\in {{\mathbb{R}}}^{C}$ is the ground truth one-hot label, ${\hat{{\bf{y}}}}_{j}\in {{\mathbb{R}}}^{C}$ the network softmax prediction, and ${\boldsymbol{\alpha }}\in {{\mathbb{R}}}^{C}$ the class weighting to encounter a class imbalance. The cross-entropy loss is averaged over a mini-batch of the size *N*. The loss function (equation (1)) is minimized by using the RAdam optimizer (Liu *et al* 2020).

For training of ECG-DualNet(++) , we utilize the Icentia11k dataset and the combined samples of datasets *A* ∪ *B* (PhysioNet/CinC 2017). We pre-train ECG-DualNet for 20 epochs on the Icentia11k dataset. The pre-trained model is then fine-tuned on the PhysioNet/CinC 2017 dataset for 100 epochs. Dependent on the task (two or four-class classification), we utilize the PhysioNet/CinC 2017 dataset with two [‘NSR’,‘AFib’] or four classes [‘NSR’,‘AFib’,‘Other’,‘Noisy’].

For training of ECG-RCLSTM-Net, we mostly used the Icentia11k dataset, from which we extracted 35 000 about sixty seconds long recordings for each label. These were then split into pretrain and test subsets with disjoint patients. The ResNet is pretrained on the pretrain subset of the Icentia11k dataset using a 20% validation split for model selection and fine-tuned on the PhysioNet dataset (*A* ∪ *B*). The CLSTM is trained in two stages. First, the CNN is pretrained on the MIT-BIH Arrhythmia Database to learn a feature representation. Then the complete CLSTM is trained on the PhysioNet dataset. Finally, ECG-RCLSTM-Net is trained using the fully trained ResNet and CLSTM and only tuning the fully connected classification layers. Here, we also use the full PhysioNet dataset and train for a single epoch.

#### 2.3.4. Implementation details

We implemented both networks using PyTorch (Paszke *et al* 2019). For implementing the preprocessing, including the data augmentation pipeline, SciPy (Virtanen *et al* 2020), NumPy (Harris *et al* 2020), and Torchaudio^9^ 
^9^

https://github.com/pytorch/audio. is used, in addition. ECG-DualNet (XL) is pre-trained on the Icentia11k dataset for 20 epochs with a batch size of 100. Pre-training on a single Nvidia Tesla V100 (32 GB) took approximately one day. Fine-tuning on the PhysioNet/CinC 2017 dataset for 100 epochs with a batch size of 24 took three hours on a single Nvidia 2080 Ti. The initial learning rate of the RAdam optimiser was 10^−3^ for both the pre-training and the fine-tuning. The learning rate was decreased after 25%, 50%, and 75% of the training (pre-training and fine-tuning) by 0.1. The first and second-order momentum factors were set to 0.9 and 0.999, respectively. Each augmentation was applied with a probability of 0.2.

### 2.4. Scoring metrics

For the evaluation, similar metrics as in the 2017 CinC challenge were used. Specifically, we used two metrics to score the submissions of the participants and our final models. Both scores were provided and visualized in a table to generate ongoing insights about the performance of each team during the 3 months period. The ranking based on *F*_1_ was visible to participants only

\begin{eqnarray*}{F}_{1}=\displaystyle \frac{\mathrm{TP}}{\mathrm{TP}+\tfrac{1}{2}(\mathrm{FP}+\mathrm{FN})},\end{eqnarray*}

where TP is the number of recordings correctly labeled ‘AFib’ , FP is the number of recordings that are labeled as ‘AFib’ for which the ground truth is ‘NSR’, FN is the number of recordings labeled as ‘NSR’ whereby ground truth is ‘AFib’. In addition, we compute the multilabel score

\begin{eqnarray*}{F}_{1,\mathrm{macro}}=\displaystyle \frac{1}{4}\cdot \sum _{i=1}^{4}\displaystyle \frac{{\mathrm{TP}}_{i}}{{\mathrm{TP}}_{i}+\tfrac{1}{2}({\mathrm{FP}}_{i}+{\mathrm{FN}}_{i})},\end{eqnarray*}

where TP_*i*_ is the number of correctly labeled recordings of class *i*, FP_*i*_ is the number of all recordings that are incorrectly labeled as class *i*, FN_*i*_ the number of all members of class *i* that were not labeled as *i*.

All unlabeled recordings were scored as if they were labeled as ‘NSR’. For *F*_1_, only recordings with ground-truth ‘NSR’ and ‘AFib’ were evaluated and predicted labels [‘Other’, ‘Noisy’] were relabeled as ‘NSR’. Besides the ranking, each team was notified about code execution and raised warnings were shared.

The final score of the the Physionet Challenge 2017 used as external validation was given as

\begin{eqnarray*}{F}_{1,\mathrm{CinC}}=\displaystyle \frac{{F}_{1,\mathrm{NSR}}+{F}_{1,\mathrm{AFib}}+{F}_{1,\mathrm{Other}}}{3},\end{eqnarray*}

where *F*_1,NSR_ is the F1 from equation (2), but with ‘NSR’ as the positive class and so on.

## 3. Results

We show the validation procedure for our models, results for the internal ranking based on *F*_1_ and *F*_1,macro_ scores as well as external evaluation on the hidden PhysioNet/CinC 2017 challenge test set. We compute the *F*_1,CinC_ for the PhysioNet/CinC 2017 challenge setting to compare ECG-DualNet++ and ECG-RCLSTM-Net with recent deep learning approaches.

### 3.1. Preliminary deep learning results

To compare the model sub-architectures of ECG-RCLSTM-Net we performed a four fold cross validation on the Physionet dataset. The ResNet alone achieves a mean *F*_1_ of 0.947, which outperforms the CLSTM with a score of 0.929. This leads to the conclusion that the ResNet is superior to the CLSTM with regards to performance. In contrast, the ResNet is with about 30 million parameters a significantly larger model than the CLSTM with about 500 000 parameters. The combined model yields the same performance on the Physionet dataset as the ResNet alone, but shows better results on the Incentia11k validation set, which hints to a better generalization capability of the overall model.

Preliminary experiments were performed to analyse the effect of the architecture choice (ECG-DualNet versus ECG-DualNet++) and the network size on the classification accuracy. In particular, we varied the width and the depth of the signal encoder. For the spectrogram encoder, we diversified the width. For ECG-DualNet we utilized four different sizes (S, M, L, and XL). The ECG-DualNet++ architecture was employed in five different sizes (S, M, L, XL, and 130 M). The preliminary results concluded that the ECG-DualNet architecture performs on par or slightly better than the advanced ECG-DualNet++ architecture in classification accuracy (F1 score). This performance gap between the ECG-DualNet and attention-based (Transformer and Axial-Attention) ECG-DualNet++ may be caused by the limited available data in dataset (*A* ∪ *B*). In terms of networks size, ECG-DualNet XL outperformed all smaller counterparts. Thus, ECG-DualNet XL is further considered. All preliminary results, trained models and an overview of all hyperparameters can be found at https://github.com/ChristophReich1996/ECG_Classification.

### 3.2. Internal ranking

All teams achieved competitive results in the binary classification setting for almost all datasets (table 2). This is especially true for test set **D** which is both out of distribution from the training set and unknown to participants and models. The superb results for validation set **B** for some teams stem from overfitting to the set, which is publicly available.

**Table 2.** Final ranking of the AI in Med. Challenge sorted by *F*_1_ Set **D** which is also the final score.

Pos.	Model name	*F*_1_ Set B	*F*_1_ Set C	*F*_**1**_ **Set D**	*F*_1,macro_ Set B	*F*_1,macro_ Set C
1	ECG-RCLSTM-Net	0.986	0.977	**0.911**	0.887	0.576
2	ECG-DualNet++	0.939	0.963	0.906	0.831	0.566
3	〈Unnamed〉	**1.000**	0.949	0.881	**1.000**	0.459
4	〈Unnamed〉	0.935	0.914	0.867	0.878	**0.598**
5	〈Unnamed〉	0.779	0.938	0.803	0.330	0.465
6	〈Unnamed〉	0.894	**0.989**	0.725	0.896	0.393
7	〈Unnamed〉	0.993	0.935	0.554	0.367	0.464

Mean		0.932	0.952	0.806	0.741	0.503
Std		0.072	0.024	0.119	0.253	0.071

The F1-Scores for the validation set **C** were very good on average with low standard deviation between teams, which can be explained by set **C** being less noisy. Teams that overfit on validation set **B**, teams that used pretraining on different openly available data, and those that only used the training data provided by us performed well on dataset **C**, which is an indicator for relatively good generalization of most models. All but two teams tried to optimize the multilabel score but did not put the same effort to the task, as can be seen when looking at the difference between teams that have good scores on *F*_1,macro_ Set **B** as opposed to *F*_1,macro_ Set **C**.

ECG-RCLSTM-Net and ECG-DualNet++ were also tested on the external PhysioNet/CinC 2017 challenge test dataset **E** to verify the results we received from our course set-up. Besides *F*_1_ we included AUROC, AUPRC, Accuracy using sklearn^10^ 
^10^

https://scikit-learn.org/stable/index.html. for computation. In table 3 both approaches show similar results to the ranking.

**Table 3.** Binary classification results on the hidden PhysioNet/CinC 2017 challenge test dataset (Clifford *et al* 2017) (**E**).

Method	*F*_1,CinC_ ( ↑ )	AUROC ( ↑ )	AUPRC ( ↑ )	Accuracy ( ↑ )
ECG-RCLSTM-Net	0.9127	0.9939	0.9623	0.9805
ECG-DualNet	0.9072	0.9901	0.9393	0.9794

### 3.3. Comparison to PhysioNet challenge 2017

After fine-tuning ECG-RCLSTM-Net and ECG-DualNet to the 4 class problem we submitted both to the official PhysioNet/CinC 2017 challenge test set (dataset **E**) to obtain the official PhysioNet Challenge Score *F*_1,CinC_. Both models performed competitively in the 4 class setting of the 2017 challenge^11^ 
^11^
Official Phase Scores https://physionet.org/content/challenge-2017/1.0.0/results all F1 scores for each classification type.csv. as can be seen from table 4 and the comparison of recent deep learning models summarized in Hong *et al* (2019). Teijeiro *et al* (2018) ranked first in the official phase of the 2017 challenge.

**Table 4.** Numerical results on the hidden PhysioNet/CinC 2017 challenge test dataset (Clifford *et al* 2017). Macro AUROC, macro AUPR and accuracy are computed with regard to all 4 classes using the sklearn implementations. We compare our models to the best models of the challenge and the mean and standard deviation (std) of the best 15 models (of a total of 67 scored models) of the challenge, as well as recent results of deep learning models summarized in Hong *et al* (2019) (— not known).

Method	*F*_1,CinC_ ( ↑ )	AUROC macro ( ↑ )	AUPRC macro ( ↑ )	Accuracy ( ↑ )
Teijeiro *et al* (2018)	0.831	—	—	—
Datta *et al* (2017)	0.829	—	—	—
Zabihi *et al* (2017)	0.826	—	—	—
Hong *et al* (2017)	0.825	—	—	—
ECG-RCLSTM-Net (ours)	0.8240	0.9453	0.8257	0.8554
ECG-DualNet (ours)	0.8003	0.9508	0.8322	0.8308

Top 15 mean (Challenge)	0.8180	—	—	—
Top 15 std (Challenge)	0.0086	—	—	—

Top 15 mean (Deep Learning) (Hong *et al* 2019)	0.8006	—	—	—
Top 15 std (Deep Learning) (Hong *et al* 2019)	0.0370	—	—	—

## 4. Discussion

The in tables 3 and 4 presented results for the selected deep learning models are close to/well above the average top scoring deep learning approaches published after the official phase of the PhysioNet/CinC challenge 2017 and the follow-up. Better performing models based on *F*_1,CinC_ rely on similar deep learning methods such as convolutional neural networks (Plesinger *et al* 2018) and LSTMs (Teijeiro *et al* 2018). Most importantly, they use extensive medical background knowledge about the physiological aspects of the ECG and hand-crafted features that reflect this, as well as relabeling of the training data by experts. Pure deep learning approaches (Zihlmann *et al* 2017, Warrick and Homsi 2018) report similar results as ours for the challenge dataset. Both presented model architectures and training procedures result in competitive performance across a wide range of datasets (**C**, **D**, **E**) from different ECG sources.

Furthermore, we observed that the pretraining accelerates the training time and the performance on the internal Icentia11k validation set but does not lead to a major difference in performance on our PhysioNet validation set.

### 4.1. Common themes in participants’ approaches

Most teams used some form of *pre-training* as a technique to train large scale models. Often, the freely available Icentia11k dataset (Tan *et al* 2019) was used. Also, the design of end-to-end deep learning models from scratch and the extensive use of preprocessing and data augmentation were common themes. Only four teams used either *dual models with hand-crafted features* (2/7) or models *purely relying on hand-crafted features* (2/7). The use of at least one *separate validation set* (7/7) generated from their data was employed by all teams, even though one team used all data at hand to train their final submission. To compare their models or check for overfitting, three teams used some kind of *cross-validation* (3/7). *CNNs* were used by almost all teams for some part of their models and four teams used *ResNet* (4/7) architectures. *Ensemble methods* (4/7) were used by four teams. They either trained two different models and performed *averaging/voting* (3/7) of the end results, or partly *trained different models together* (1/7). A *spectrogram* (3/7) of the ECG was used as input for the classifiers of three teams. *Data augmentation methods* (4/7) were used by four teams and all found that this improved the performance of their respective models significantly. Interestingly, some teams saw improvements from using ensemble models while others did not. Four teams tried *additional datasets* (4/7) for pretraining. However, they saw no improvements in *F*_1_ when training and validating on the CinC 2017 dataset.

**Figure 5.**
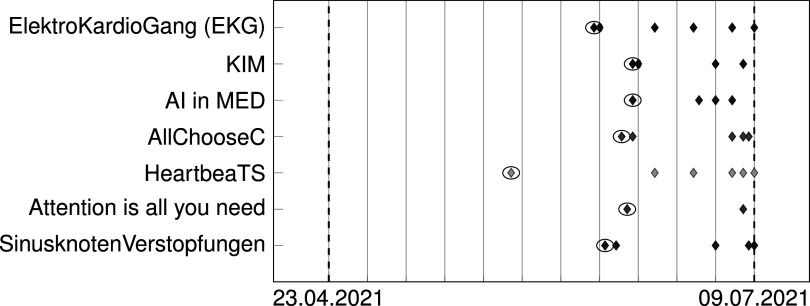
Submissions per Team plotted from kick-off to final submission. Vertical lines indicate weeks. The encircled submissions are first attempted submissions, that not necessarily ran successfully on our server, but indicate the first try of each team.

Another interesting yet expected outcome can be seen in figure 5: as with the original challenge, submissions clustered at the end of the validation period, which lead us to implement improvements for the course. In particular, we imposed a mandatory midterm presentation and introduced fixed, overlapping time windows for each of the 5 possible submissions. Both measures were taken in order to distribute the workload more evenly over the whole semester and thus lessen the pressure for the final week and improving the overall (median) performance of all teams, recognizing that top teams might not be affected. Having deadlines for all 5 submissions showed no improvement in student engagement, while also having no benefits regarding submission clustering towards the end of the course compared to a single mandatory and early first submission.

### 4.2. Participant self-assessment

**Figure 6.**
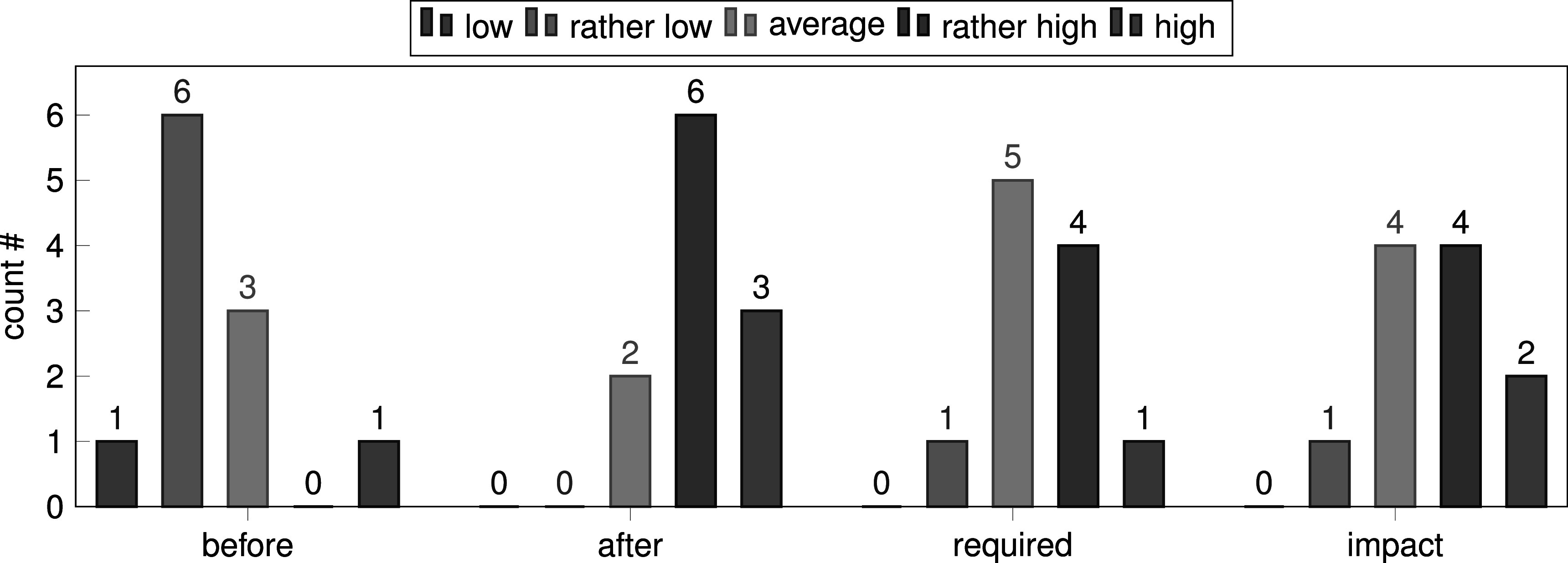
The plot shows the self-assessed knowledge in machine learning and ECG-Analysis of the students before and after the course as well as the perceived required knowledge for participating and the impact of the course on knowledge-gain.

**Figure 7.**
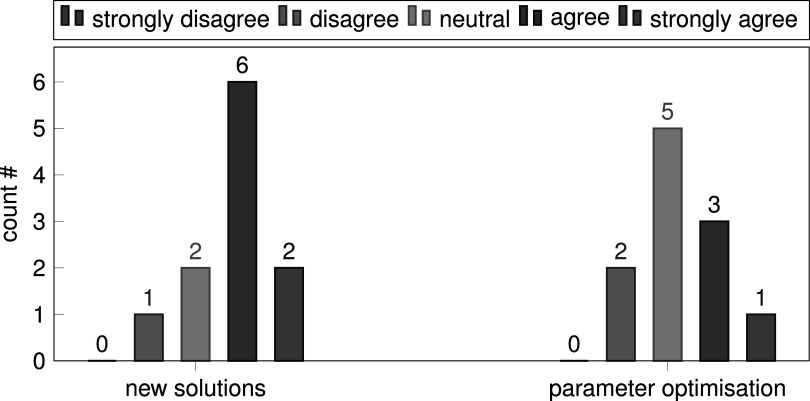
‘The leaderboard of the challenge motivated students to focus on...’

We designed a short survey which was taken anonymously after the final grade was assigned (*n* = 11). It contained questions about the perceived impact of the course on the methodological knowledge of the participants. In addition, we asked about the impact of the leaderboard on specific motivation. Figure 6 shows that the most common answer in terms of knowledge before and after the course changed from ‘rather low’ to ‘rather high’. Interestingly, the majority of the participants rated both the requirements on prior knowledge for the course and the impact of the course on knowledge gain as ‘average’ and ‘rather high’. The leaderboard probably motivated the participants far more to try new methods than it did to perform parameter tuning as shown by figure 7, which we believe is an encouraging result. Nine participants selected interest in AI and Machine Learning as the main reason for participating in our class while a good time/credit point ratio and interest in teamwork were selected once each. On the other hand, interest in medicine seems to be only a minor reason. Thus, both prior knowledge in medicine and machine learning of the participants were rather low in the beginning of the Challenge. We learned from the free-text responses that participants particularly liked the fact that there were only few restrictions regarding code requirements. They also appreciated that working on the same task led to seeing multiple solutions. We found it somewhat surprising that students actually suggested to impose more restrictions by introducing mandatory submissions during the course and a mandatory halftime presentation (which we did in the next iteration of the class). Finally, the students also addressed environmental aspects of machine learning by proposing limits on computation-time and dataset-size.

### 4.3. Peculiarities of our challenge

We set up our own evaluation system for this course which will be shared as an easy-to-use set of scripts^12^ 
^12^
Evaluation system: https://github.com/KISMED-TUDa/ai_med_evaluation.. As a matter of fact, multiple systems exist that support evaluation and set up of competitions. The main drawbacks of using externally hosted competitions are privacy concerns and the compulsion to share datasets with the hosts. Codalab^13^ 
^13^

https://github.com/codalab/codalab-competitions. provides a well-documented, flexible system, that is open source and thus can be hosted on own servers and might be a good alternative to using our evaluation system, while only adding some complexity. Also, the semi-automatic analysis method which was intended to lower the threshold for beginners compared to a fully automated system tends to result in a significant overhead even for this small cohort. The main reason for that is not the evaluation system itself but the lack of direct and automatic feedback about successful runs. Therefore, we plan to switch to a Jupyter-Notebook-based evaluation system, which reduces overhead on both participants and supervisor and benefits the participants additionally by creating a separate learning environment.

## 5. Conclusion

The two best models of the course were validated against the PhysioNet/CinC Challenge 2017 test set for both *F*_1_ and official challenge score and achieved near state-of-the-art results. Although these results were close in performance to the top scoring models, it must be emphasized that the existing difference is ultimately relevant for the application in patient monitoring. Additionally, we showed that the PhysioNet/CinC Challenge of 2017 provides a suitable platform for educating master level engineering students in machine learning for biomedical tasks. While originality and good analysis of the models were most relevant for grading and emphasized regularly, the competitive nature of the course lead to objectively competitive models. The high scores as shown in the ranking and individual statements by participants on the voluntary but high workload demonstrate known aspects of gamification. All teams employed well known and recent methods based on hand-crafted features and/or deep learning to engineer competitive models. In the following semester, we added mandatory mid-term submissions which mimic the unofficial/official phase of the CinC challenge and increased emphasis on the mid-term presentation based on student suggestions. First results imply a decrease in the early model scores with a faster increase between submissions compared to the first iteration of the course. We provide links and documentation to our example code and evaluation system.
